# Obtaining History with a Language Barrier in the Emergency Department: Perhaps not a Barrier After All

**DOI:** 10.5811/westjem.2018.8.39146

**Published:** 2018-09-10

**Authors:** Megan Litzau, Joseph Turner, Katie Pettit, Zachary Morgan, Dylan Cooper

**Affiliations:** Indiana University, Department of Emergency Medicine, Indianapolis, Indiana

## Abstract

**Introduction:**

Patients with limited English proficiency may be at risk for incomplete history collection, potentially a patient safety issue. While federal law requires qualified medical interpreters be provided for these patients, little is known about the quality of information obtained in these encounters. Our study compared the medical histories obtained by physicians in the emergency department (ED) based on whether the patients primarily spoke English or Spanish.

**Methods:**

This was a prospective, observational study conducted at a single, urban, academic ED during a six-month time period. Resident and faculty physicians caring for adult patients with a chief complaint of chest or abdominal pain were eligible for participation. Patient encounters were directly observed by medical students who had been trained using simulated encounters. Observers documented which key historical data points were obtained by providers, including descriptions of pain (location, quality, severity, radiation, alleviating/aggravating factors), past medical/family/surgical history, and social history, in addition to the patient’s language in providing history. Providers, interpreters, and observers were blinded to the nature of the study. We used chi-square analyses to examine differences in whether specific elements were collected based on the primary language of the patient.

**Results:**

Encounters with 753 patients were observed: 105 Spanish speaking and 648 English speaking. Chi-square analyses found no statistically significant differences in any history questions between Spanish-speaking and English-speaking patients, with the exception that questions regarding alleviating factors were asked more often with Spanish-speaking patients (45%) than English-speaking patients (30%, p=.003). The average percentages of targeted history elements obtained in Spanish and English encounters were 60% and 57%, respectively.

**Conclusion:**

In this study at a large, urban, academic ED, the medical histories obtained by physicians were similar between English-speaking and Spanish-speaking patients. This suggests that the physicians sought to obtain medical histories at the same level of detail despite the language barrier. One limitation to consider is the Hawthorne effect; however, providers and observers were blinded to the nature of the study in an attempt to minimize the effect.

## INTRODUCTION

Patients with limited English proficiency experience a disproportionate number of adverse events.^1^ Under Title VI of the United States (U.S.) Civil Rights Law of 1964, healthcare institutions receiving federal funding are prohibited from discriminating against patients of limited English proficiency.^2^ More recently, the Affordable Care Act Section 1557 requires that all healthcare institutions receiving federal funds provide qualified medical interpreters to patients of limited English proficiency.^3^ While federal law requires qualified medical interpreters be provided for these patients, little is known about the quality of information that is obtained in these encounters. To our knowledge, there are no studies comparing the medical histories obtained in English- vs. Spanish-speaking patients in the emergency department (ED). It was against this background that our study was designed to compare the medical histories obtained by emergency physicians based on whether patients primarily spoke English or Spanish. We hypothesized that due to an increased time requirement required for interpretation, providers on average would ask fewer questions of Spanish-speaking patients.

## METHODS

### Design and Setting

This was a prospective observational study conducted at an urban, teaching hospital ED with approximately 100,000 patient visits annually. Data was collected from February 2017 through July 2017. The study was approved by the institutional review board.

### Study Procedure and Participants

Study investigators created a checklist to assess completeness of history that providers obtained. The checklist contained 12 historical items of interest: six items pertaining to the history of present illness (HPI), as well as past medical history, past surgical history, family history, social history, education, and allergies. The checklist also contained patient demographic information, a question about the language used for the patient encounter, and additional data points designed to keep all observers and participants blinded to the nature of the study. The question about encounter language had four answer options: English, Spanish without interpretation (provider spoke Spanish), Spanish with formal interpreter, and Spanish with family interpretation. The full checklist can be found in the Appendix.

Volunteer medical students served as observers. Observers were trained by study investigators to navigate the ED and to use the data collection checklist. Following initial training, observers viewed simulated patient encounters and recorded the interactions using the data collection checklist. Study investigators reviewed the training scores to ensure adequacy of training. Observers were kept blind to the nature of the study and outcomes of interest. Observers followed emergency medicine (EM) residents and faculty during their shifts. All EM residents and faculty working in the ED participated in the study. Observer shift times were scheduled according to observer availability but included a variety of morning, afternoon, evening, and overnight shifts as well as both weekday and weekend shifts.

During shifts, the volunteers observed provider-patient encounters with a chief complaint of chest pain or abdominal pain. They obtained verbal consent from the patient to witness the initial history and physical exam encounter. Subsequently, they continued to follow the provider throughout the rest of the provider’s care of that patient, including diagnostic and treatment management, any performed procedures, and dispositioning the patient. This was done so that participants would remain blind to the nature of the study. The student observers also collected additional information including elements of the physical exam and orders that were placed for the patients including laboratory, radiology and medication orders to maintain blindness. Observers recorded all checklist items that were performed throughout the encounter in real time using tablets and the Research Electronic Data Capture (REDCap) database. REDCap is a secure, web-based application designed to support data capture for research studies.^4^

### Outcome Measures

The primary outcome of interest was the percentage of HPI items obtained. With a sample size of 500 patients we calculated 99.4% power to detect an average of half a question difference at alpha = 0.05. Secondary outcomes included whether or not each of the 12 individual historical questions were obtained. While additional data points were primarily used for blinding purposes, we analyzed the data for any differences in performance of physical exam and the workup/treatment that was performed.

## RESULTS

During the six-month data collection period, 753 patient encounters were observed. Of those encounters, 105 patients spoke Spanish and 648 spoke English. Chi-square analyses found no statistically significant difference in any of the history questions between the Spanish-speaking and English-speaking groups with the exception of alleviating factors. The question of alleviating factors was asked more often with Spanish-speaking patients (45%) than English-speaking patients (30%, p=.003) (Table 1). The average percentages of targeted history elements that were obtained in Spanish and English encounters were 60% and 57%, respectively. Table 2 displays result by translator type.

## DISCUSSION

Patients with limited English proficiency represent a vulnerable patient population in our healthcare system. There are multiple variables in patient care that could lead to inequitable outcomes. In EDs, the initial patient encounter including the history and physical exam is crucial for downstream patient care. For this reason, we decided to examine the difference in the history obtained between our English- and Spanish-speaking patients.

Our initial hypothesis that providers are not as thorough or detail oriented in their history taking with Spanish-speaking patients was not supported in this study. In fact, the only historical component that attained statistical significance (alleviating factors) favored the Spanish-speaking patients. Although unexpected, this is a reassuring finding.

Since this was not expected, we considered possibilities that would lead to this finding. One is that providers wanted to take advantage of the time they had with the interpreter. They may have been asking all questions that could possibly be applicable during that initial encounter, as they knew that getting additional clarification later might have been difficult. Another possibility is that the institution used in this study has extremely proficient and available interpreters. These findings may not hold true at other institutions with a variety of available language services. Finally, perhaps our providers are aware of the vulnerability of this patient population and actively focus on thorough histories as a safety mechanism. Nonetheless, the evidence still points to healthcare disparities in this patient population. If the disparity doesn’t lie in history taking, we need to examine other variables in patient care.

## LIMITATIONS

This study was conducted at a single, academic, tertiary-care site in a Midwestern city in the U.S. The patient population primarily speaks English with the second most common language Spanish. As such, we only evaluated patient encounters using these two languages. An additional limitation of the study is the Hawthorne effect. We tried to control for this by blinding both the medical student observers and the residents and faculty who were being observed to the purpose of the study; however, the mere presence of the observer could have significantly altered the provider’s history taking.

## CONCLUSION

In this study at a large, urban, academic ED, the medical histories obtained by physicians were similar between English-speaking and Spanish-speaking patients. This suggests that the physicians sought to obtain medical histories at the same level of detail despite the language barrier. In some instances, the trend was toward more history obtained in the Spanish-speaking patients vs. the English-speaking patients. Areas for future study include noting the amount of time spent in the room with Spanish-speaking vs. English-speaking populations, evaluating the histories obtained by residents and by faculty, and evaluating different interpreter modalities including phones, video, and live interpretation.

## Supplementary Information



## Figures and Tables

**Table 1:** Elements of patient encounter obgtained: English compared to Spanish.

		Language				
			
		English		Spanish		
				
		N	%	N	%	Chi-Square
History of present illness (HPI) (choice=location)	No	24	3.7%	2	1.9%	0.877
	Yes	624	96.3%	103	98.1%	0.349
History of present illness (HPI) (choice=quality)	No	133	20.5%	25	23.8%	0.588
	Yes	515	79.5%	80	76.2%	0.443
History of present illness (HPI) (choice=severity)	No	436	67.3%	72	68.6%	0.068
	Yes	212	32.7%	33	31.4%	0.794
History of present illness (HPI) (choice=radiation)	No	280	43.2%	41	39.0%	0.640
	Yes	368	56.8%	64	61.0%	0.424
History of present illness (HPI) (choice=alleviating factors)	No	453	69.9%	58	55.2%	8.915
	Yes	195	30.1%	47	44.8%	0.003*
History of present illness (HPI) (choice=aggravating factors)	No	349	53.9%	53	50.5%	0.415
	Yes	299	46.1%	52	49.5%	0.519
Additional history (choice=past medical history)	No	36	5.6%	6	5.7%	0.004
	Yes	612	94.4%	99	94.3%	0.948
Additional history (choice=surgical history)	No	343	52.9%	61	58.1%	0.969
	Yes	305	47.1%	44	41.9%	0.325
Additional history (choice=family history)	No	546	84.3%	85	81.0%	0.728
	Yes	102	15.7%	20	19.0%	0.394
Additional history (choice=social history)	No	252	38.9%	48	45.7%	1.756
	Yes	396	61.1%	57	54.3%	0.185
Medications (choice=medications)	No	155	23.9%	28	26.7%	0.371
	Yes	493	76.1%	77	73.3%	0.543
Medications (choice=allergies)	No	418	64.5%	71	67.6%	0.385
	Yes	230	35.5%	34	32.4%	0.535

* Statistically significant result.

**Table 2:** History elements obtained by language spoken and type of translator used.

	Spanish			English	Chi-square	Significance

	Family	Interpreter	Provider			
Location	100.0%	97.5%	96.3%			
Quality	81.8%	78.5%	79.5%			
Severity	54.5%	29.1%	32.7%	26.7%	3.09	0.377
Radiation	72.7%	57.0%	56.8%	73.3%	2.72	0.437
Alleviating factors	63.6%	43.0%	30.1%	40.0%	10.98	0.012
Aggravating factors	54.5%	49.4%	46.1%	46.7%	0.58	0.902
Past medical history	100.0%	94.9%	94.4%	86.7%	2.4	0.493
Surgical history	63.6%	41.8%	47.1%	26.7%	4.46	0.216
Family history	9.1%	19.0%	15.7%	26.7%	2.17	0.537
Social history	45.5%	55.7%	61.1%	53.3%	2.19	0.535

Percentage of time questions were asked in history of present illness.
