# Supplementary material for: Obtaining History with a Language Barrier in the Emergency Department: Perhaps not a Barrier After All
Source: West J Emerg Med. 2018 Sep 10;19(6):934–7. doi: 10.5811/westjem.2018.8.39146 (PMC6225939; doi:10.5811/westjem.2018.8.39146)
Supplement: Supplementary file 1 [file wjem-19-934-s001.docx]

**APPENDIX**

| Gender | Male  Female |
| --- | --- |
| Age | 0-19  20-29  30-39  40-49  50-59  60-69  70-79  Over 80 |
| Language | English  Spanish-interpreter  Spanish-provider  Spanish-family member |
| HPI | Location  Quality  Severity  Radiation  Alleviating Factors  Aggravating Factors |
| Additional history | Past medical history  Surgical history  Family history  Social history |
| Medications | Medications  Allergies |
| Physical Exam | Auscultation of chest  Palpation of chest wall  Evaluate for peripheral edema  Auscultation of abdomen  Palpation of abdomen |
| Work up | Urine  Blood  Plain film XR  US (Formal or bedside)  CT scan  Narcotic pain medication  Non narcotic pain medication  Nitro  Aspirin  Antiemetics |
